# Asymmetric Manipulation of Perpendicular Exchange Bias and Programmable Spin Logical Cells by Spin–Orbit Torque in a Ferromagnet/Antiferromagnet System

**DOI:** 10.1002/advs.202403648

**Published:** 2024-07-10

**Authors:** Lei Guo, Guopeng Shi, Guocai Wang, Hua Su, Huaiwu Zhang, Xiaoli Tang

**Affiliations:** ^1^ State Key Laboratory of Electronic Thin Films and Integrated Devices School of Electronic Science and Engineering University of Electronic Science and Technology of China 22006 Xiyuan Avenue, High‐tech Zone (West) Chengdu Sichuan 611731 China

**Keywords:** antiferromagnet (AFM), asymmetry, exchange bias, spin logic, spin‐orbit torque (SOT)

## Abstract

Antiferromagnets are competitive candidates for the next generation of spintronic devices owing to their superiority in small‐scale and low‐power‐consumption devices. The electrical manipulation of the magnetization and exchange bias (EB) driven by spin‐orbit torque (SOT) in ferromagnetic (FM)/antiferromagnetic (AFM) systems has become focused in spintronics. Here, the realization of a large perpendicular EB field in Co/IrMn and the effective manipulation of the magnetic moments of the magnetic Co layer and EB field by SOT in Pt/Co/IrMn system is reported. During the SOT‐driven switching process, an asymmetrically manipulated state is observed. Current pulses with the same amplitude but opposite directions induce different magnetization states. Magneto–optical Kerr measurements reveal that this is due to the coexistence of stable and metastable antiferromagnetic domains in the AFM. Exploiting the asymmetric properties of these FM/AFM structures, five spin logic gates, namely AND, OR, NOR, NAND, and NOT, are realized in a single cell via SOT. This study provides an insight into the special ability of SOT on AFMs and also paves an avenue to construct the logic‐in‐memory and neuromorphic computing cells based on the AFM spintronic system.

## Graphical abstract

Asymmetric manipulation of perpendicular exchange bias (EB) by Spin–Orbit Torque (SOT) in a ferromagnet (FM)/antiferromagnet (AFM) system is developed. The coexistence of stable and metastable antiferromagnetic magnetic domains leads to the different magnetization states induced by current pulses with the same amplitude but in opposite directions. Five logic gate units are realized via the asymmetric properties.

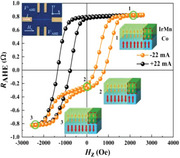

## 1 Introduction

Electrical manipulation of the magnetization in magnetic films has attracted tremendous attention for the next generation of spintronic applications. It has great potential for achieving the high‐performance and low‐power consumption devices. The Spin‐Orbit Torque (SOT), which is generated by heavy metals (HMs), such as Pt, Ta, and W, is an efficient approach to attain electrical manipulation. Not only ferromagnetic (FM) films but also antiferromagnetic (AFM) films can be modulated by SOT.^[^ ^1^, ^2^, ^3^, ^4^, ^5^ ^]^ AFM material is a special kind of magnetic material. Their magnetic order is staggered, and their net magnetic moment is zero. Owing to its unique features, such as ultrafast spin dynamics, robustness against an external field, and absence of macroscopic magnetization,^[^ ^6^, ^7^ ^]^ AFM materials show numerous advantages in novel spintronic applications.

In recent years, an increasing number of studies have focused on electrical manipulation, especially adopting the SOT effect, to modulate the AFM moments. The ability to control AFM moments paves a new avenue for the realization of multifunctional and flexible AFM spintronic devices, which will be useful for information storage and neuromorphic computing, and so on.^[^ ^8^, ^9^ ^]^ The AFM state of antiferromagnetic semimetals or semiconductors like Mn_3_Sn and NiPS_3_ can be modulated by controlling the topological spin textures of AFMs.^[^ ^10^, ^11^, ^12^ ^]^ Alternatively, the exchange bias (EB) can be tuned by adjusting the interfacial spins in normal AFMs like IrMn and FeMn.^[^ ^13^, ^14^ ^]^ The EB, originating from the FM and AFM interfacial exchange coupling, is the most important effect generated from AFM materials. In addition to being used in magnetic storage devices as read heads or memory cells, EB systems also provide a significant method to explore the variation of the FM/AFM interfacial state.^[^ ^15^ ^]^ Very recently, several studies have reported the switching of AFM interfacial order through the EB field in metallic HM/FM/AFM multilayer films by SOT.^[^ ^16^, ^17^ ^]^ These results are significant for future applications as metallic FM/AFM structures are already utilized in present spintronic devices and match CMOS process. However, findings regarding the electrical manipulation of EB structures are not completely the same. In some studies, the spin‐polarized current could switch back and forth the direction of the EB field entirely by changing the direction of the current or an in‐plane (IP) magnetic field.^[^ ^18^, ^19^ ^]^ However, in other reports, the polarized current with the IP magnetic field could only partially switch the EB field.^[^ ^20^, ^21^ ^]^ Furthermore, some studies reported that the switching was strongly related to the orientation of the neighboring FM moments.^[^ ^22^, ^23^ ^]^ Since the EB is an interfacial effect, its strength and stability are strongly correlated to the interfacial uncompensated spins and the variations in the AFM‐grain size.^[^ ^24^ ^]^ For samples with different interfacial states, they particularly affect the local magnitude of the EB field and the SOT‐driven magnetization reversal.

To decrease the impact of the interfacial unstable state, here, we selected a Pt/Co/IrMn films structure with a high perpendicular EB field (larger than 1500 Oe). By applying the SOT combined with an IP magnetic field, we can effectively modulate the AFM interfacial spins and the EB field. Moreover, an asymmetric electrical manipulation of the EB field in this relatively stable EB sample is demonstrated by the observation of an unable driven pinned region in the Hall cross and some discontinuous scattering areas in the FM/AFM structure. Furthermore, based on this asymmetric manipulating property, effective programmable spin logics are realized. Five logic gates, namely AND, OR, NAND, NOR, and NOT, are reconfigured in one EB cell. These findings open an important gateway toward the realization of practical programmable AFM spin logic cells and have potential applications in future logic‐in‐memory devices.

## 2 Results and Discussion

### 2.1 Measurement Configuration and Basic Properties of the EB System

**Figure 1:**
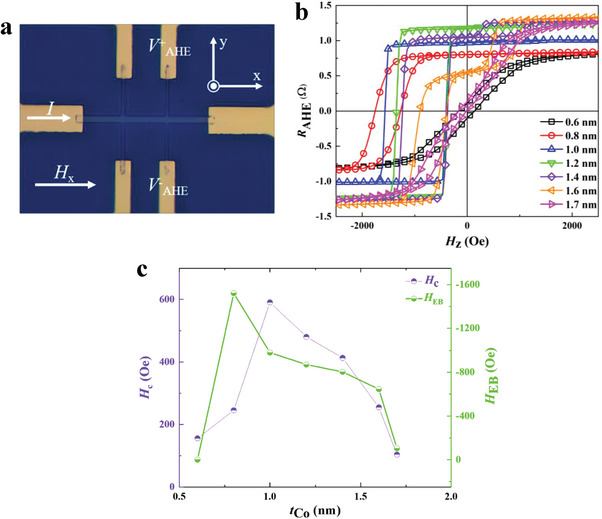
a) Micrograph of the Hall bar device for AHE measurements. *V*_AHE_ represents the anomalous Hall voltage, and the arrows signed *H*_x_ and *I* denote the directions of the IP magnetic field and current pulse respectively. b) *R*_AHE_ versus *H*_z_ curves for as‐grown samples with *t*_Co_ from 0.6 to 1.7 nm. c) The plots of *H*_EB_ and *H*_c_ versus Co thickness.

Stacked structures consisting of Ta (1 nm)/Pt (3 nm)/Co (*t*_Co_)/Ir_20_Mn_80_ (5 nm)/Ta (1.5 nm) with *t*_Co_ ranging from 0.6 to 1.7 nm were prepared. The optical micrograph of the samples and the geometry of the Anomalous Hall Effect (AHE) measurement are presented in **Figure** **1**a. The AHE resistance (*R*_AHE_) as a function of the out‐of‐plane magnetic field (*H*_z_) for the as‐deposited samples is shown in Figure 1b. The exchange bias fields (*H*_EB_) and coercive fields (*H*_c_) are shown in Figure 1c. When *t*_Co_ layer is smaller than 0.8 nm, the AHE loop does not show clear PMA features due to the weak orbital hybridization between the Co 3d and Pt 5d orbitals at the interface, and the EB effect does not appear either. As the *t*_Co_ increases to 0.8 nm, the structure exhibits outstanding PMA and a perpendicular EB field. It achieves the largest EB field (1520 Oe) among the as‐grown samples. This result is consistent with the fact that the EB field is roughly inversely proportional to the thickness of the FM layer.^[^ ^25^ ^]^

As the thickness of the Co layer *t*_Co_ reaches to 1.4 nm, a clear bidirectional EB effect appears with decreasing EB field *H*_EB_. It is commonly observed in an as‐deposited or relatively thick magnetic film with decreasing perpendicular anisotropy. This phenomenon is attributed to the coexistence of two domain populations with opposite orientations at the FM/AFM interface.^[^ ^26^, ^27^, ^28^ ^]^ In order to avoid the uncertainty of bidirectional *H*_EB_, a Co film with a thickness of 0.8 nm with a large *H*_EB_ and a strong stability was selected in the following experiments. What's more, too thick *t*_Co_ weakens the influence of the interfacial effect between Pt/Co and Co/IrMn to the magnetic moments of the Co layer, which leads to the decline of PMA and exchange bias.

### 2.2 SOT Manipulation of the Perpendicular EB Structures

The SOT is physically generated by the transfer of spin angular momentum from the HM layer to the magnetic system. It depends on the conversion efficiency of the electron current into the spin current.^[^ ^29^, ^30^ ^]^ In the present study, we mainly focus on the tunability of the AFM interfacial spins which reflects the change in the direction and magnitude of the EB field through the SOT.

**Figure 2:**
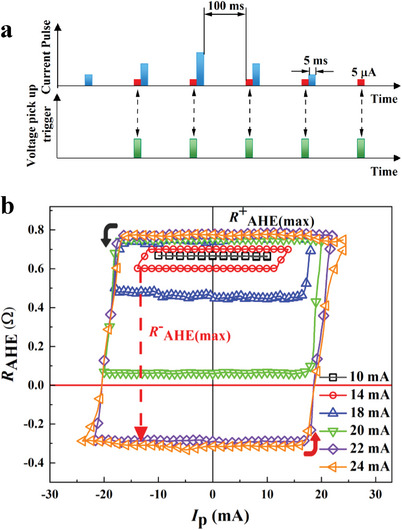
a) The method to measure dependence of *R*_AHE_ on applied current. b) *R*_AHE_ versus *I*_p_ curves with varying *I*_p_ from 10 to 24 mA and *H*_x_ = 300 Oe.

For the SOT‐driven experiments in the Ta (1 nm)/Pt (3 nm)/Co (0.8 nm)/IrMn (5 nm)/Ta (1.5 nm) system, a current *I*_p_ and an IP magnetic field *H*_x_ of 300 Oe are applied along the x‐axis. Although the underlying Ta layer is also an HM with a large spin Hall angle opposite to that of the Pt layer, its resistivity is 10 times larger than that of Pt.^[^ ^31^, ^32^, ^33^ ^]^ Therefore, its influence is ignored in the present study. To acquire detailed SOT‐driven magnetization switching loops, the ranges of *I*_p_ are changed from 10 to 24 mA. Moreover, a SOT‐driven pulse combined with a small sampling current to pick up the Hall voltage is selected to probe the stable nonvolatile magnetization state induced by the SOT effect. During the measurement, the SOT‐driven pulse is first applied and released. Then, after an interval time of 100 ms, a subsequent pulse with an amplitude of 5 µA and a duration of 5 ms is adopted to pick up the Hall voltage. This procedure is illustrated in **Figure** **2**a. The dependence of *R*_AHE_ on the applied current *I*_p_ with *H*_x_ of +300 Oe is displayed in Figure 2b (see dependence of *R*_AHE_ with different *H*_x_ in Figure S1, Supporting Information) and is similar to that of Pt/Co structure (see the *R*_AHE_ loops of Pt/Co structure in Figure S2, Supporting Information). It confirms the SOT effect dominates in the switching process. The result shows that the magnitude of *R*_AHE_ = *R* ^+^_AHE (max)_ – *R* ^−^_AHE (max)_ gradually increases upon increasing *I*_p_, and it almost saturates as *I*_p_ reaches 22 mA with a current density of 1.94 × 10^7^ A cm^−2^. Furthermore, in the loops, *R* ^+^_AHE (max)_ changes very little, while *R* ^−^_AHE (max)_ changes step by step. The results reflect the asymmetric magnetization switching states. Considering the *R*_AHE_ value in Figure 2b, we can infer that the SOT induced by the positive current can drive the magnetic moments of the Co layer to switch completely in the +z‐direction. However, a negative polarized current with the same magnitude can only switch part of the magnetic moments to the ‐*z*‐direction. Furthermore, even upon increasing *I*_p_ to −24 mA, *R* ^−^_AHE (max)_ remains almost the same as that at *I*_p _= −22 mA and becomes saturated. This behavior is completely different from the SOT‐driven magnetization switching in a single Co layer.^[^ ^34^, ^35^, ^36^ ^]^ The *R* ^±^_AHE (max)_ is completely symmetric.

**Figure 3:**
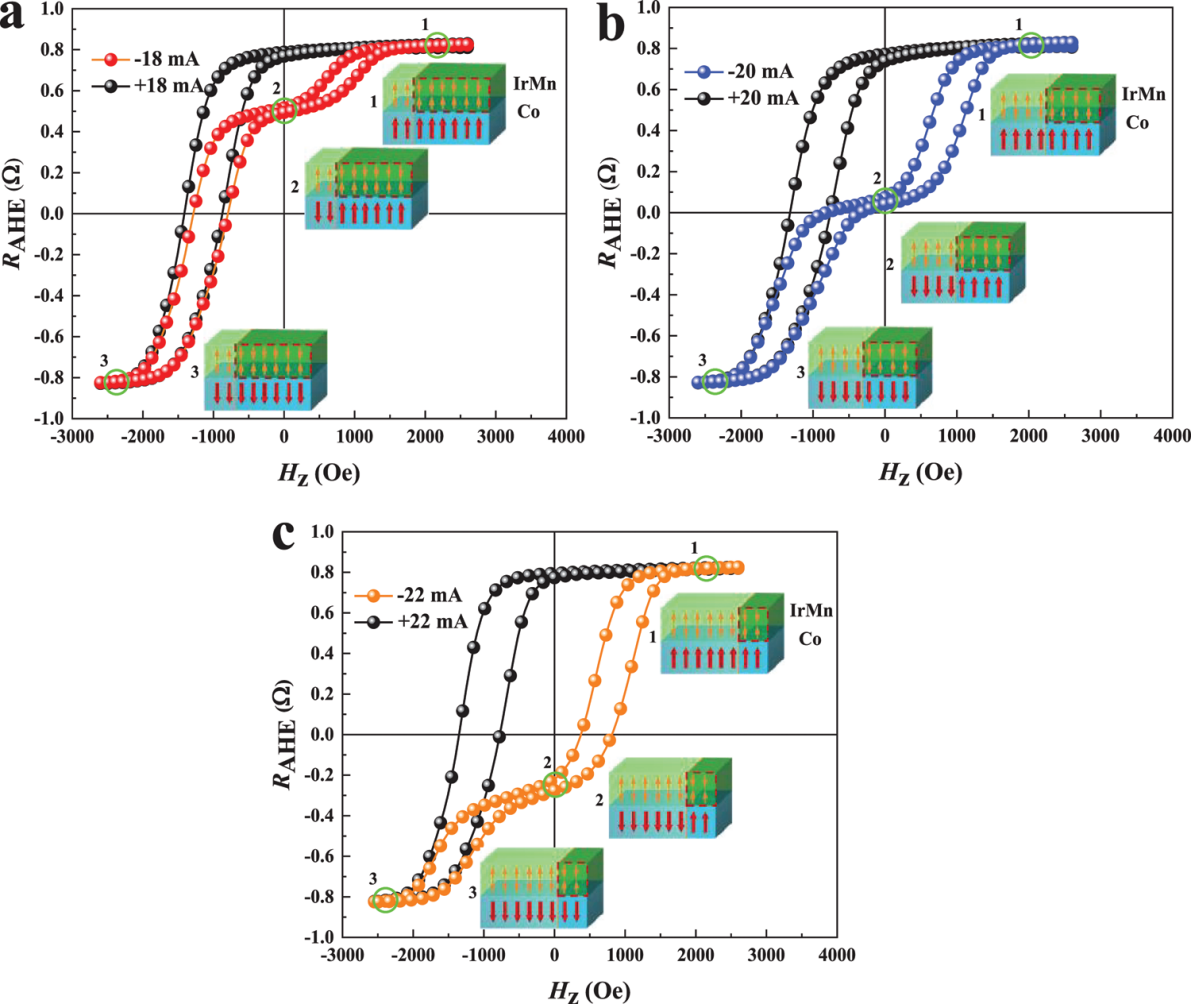
AHE loops after different current pulses with *H*_x_ = 300 Oe and schematics of the magnetic moments in Co/IrMn bilayer after the negative pulse is applied. The red dashed box represents the unable modulated AFM moments. a) *I*_p_ = ∓18 mA. b) *I*_p_ = ∓20 mA. c) *I*_p_ = ∓22 mA.

In order to further understand this asymmetric switching, we focus on the changes in the EB field and magnetization curves. The *R*_AHE_ versus *H*_z_ loops are obtained upon applying negative and positive current pulses *I*_p_. Pulse currents of ∓18 mA, ∓20 mA, and ∓22 mA are selected. During this process, the IP field *H*_x_ is set to +300 Oe with the negative pulse applied first. Subsequently, *H*_x_ is set to 0 Oe, and the *R*_AHE_ loop versus *H*_z_ loop is measured. Then, a current pulse with the same magnitude and opposite direction (positive pulse) is applied with *H*_x_. Subsequently, its *R*_AHE_‐*H*_z_ curve is measured. These two loops are plotted together as a group in **Figure** **3**. The corresponding schematics are also shown in the figure. State 1 and State 3 represent the situations, where under a sufficiently strong external magnetic field, the magnetic moment of the Co layer aligns fully with the external magnetic field, either in the positive or negative direction. While State 2 depicts the distribution of the Co layer's magnetic moment, which remains consistent with the IrMn interfacial spin in zero magnetic field. The *R*_AHE_ achieved at zero field is consistent with the asymmetric *R* ^−^_AHE (max)_ and *R* ^+^_AHE (max)_ displayed in Figure 2.

For our sample, the initial direction of the *H*_EB_ is along the +*z*‐axis, as described in Figure 1b. When a negative pulse is first applied, a two‐step hysteresis loop is observed, as shown in Figure 3. This two‐step hysteresis loop indicates that up‐ and down‐oriented AFM domains coexist at the FM/AFM interface. These AFM domains pin magnetic moments of the Co layer along two directions at zero field. An increase in the current pulse leads to a reduction in the AFM domains represented by the red rectangular box in IrMn. However, when a positive pulse with the same amplitude is applied, this two‐step hysteresis loop reverts to its initial state.

As the magnitude of the negative pulse increases, the switched proportion of the *H*_EB_ from the initial positive direction to the negative direction increases. However, the initial positive *H*_EB_ cannot be switched completely to the negative direction, even when the current pulse reaches −22 mA, which is near saturation. This means that part of the AFM moments cannot be modulated (indicated by the red dashed box), and they pin the magnetic moments of the Co layer along the initial EB direction at 0 Oe. From these findings, several important conclusions can be drawn. First, the SOT can not only modulate the FM moments but also the AFM moments and the EB field. In previous studies, it has been reported that spin‐polarized currents cannot pass through a thick FM layer. The characteristic saturation length is 1.2 nm.^[^ ^37^ ^]^ The *t*_Co_ in our present study is only 0.8 nm. This means that the spin‐polarized current generated in the Pt layer can pass through the Co layer and reach the Co/IrMn interface. Therefore, the SOT effect induced by the HM layer changes the magnetization of the Co layer and the moments of the Co/IrMn interface simultaneously. This result is consistent with recent works.^[^ ^22^, ^38^ ^]^

Second, the local arrangement of the AFM domains and the spin‐polarized current distribution may be complex. They give rise to different conditions for changing the EB effect in the same film in various regions. Therefore, by applying polarized currents with the same amplitude in opposite directions, different magnetization states are obtained, which explains the results presented in Figure 3.

The Joule heating is usually considered to be a very important issue in SOT switching process.^[^ ^39^ ^]^ To verify whether the switching at the IrMn interface is caused by the Joule heating induced by the applied current, we have achieved the temperature rise of IrMn by applying current pulses. The results are displayed in Section S3 (Supporting Information). The blocking temperature *T*_B_ for the IrMn layer in the study is approximately 440 K, which is significantly higher than the maximum temperature increase of approximately 40 K caused by the applied current of 22 mA in the experiment. Therefore, we have excluded the effect of Joule heating on the switching of the IrMn interfacial spin.

### 2.3 Magneto–Optical Kerr Measurements for Understanding the Evolution of the SOT Effect

**Figure 4:**
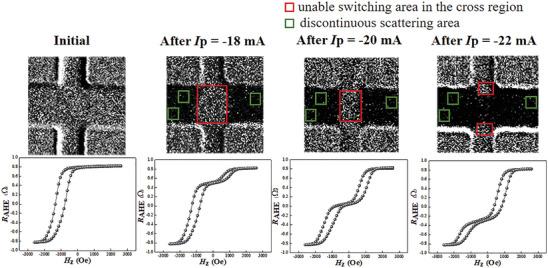
Evolution of remnant magnetic domains in Hall Bar with the current pulse changing with *H*_x_ = 300 Oe. The magnetic domain images were taken by Kerr Microscope after applying current pulses, and the corresponding hysteresis loops are also shown between the images.

To gain a deep insight into the changes in the magnetic domains in the FM/AFM EB system, magneto–optical Kerr microscopy measurements were performed on the samples. Pulses with different amplitudes were applied to achieve different EB states. During the measurements, we mainly focused on the Hall cross region as the picked‐up voltage to calculate the *R*_AHE_ is determined by the condition in the Hall cross. The evolution of the magnetic domain images and the corresponding hysteresis loops for different current pulses are displayed in **Figure** **4**. In the initial state, a completely grey area is observed. Since the initial sample has a large *H*_EB_, all the magnetic moments are pinned in the “up” state. As the amplitude of the negative current pulse increases, it is obvious that the dark grey domains (which represent the magnetization of the Co layer oriented in the “down” state) grow. The switching process starts from outside the cross region, and then spreads to the cross region. Furthermore, several discontinuously scattered grey areas can be observed in the sample (marked by green rectangles), in which the magnetic moments are oriented in the positive direction and cannot be switched by the SOT. As the current pulse increases to −22 mA, which is close to a saturating switching pulse, the grey domains are still present in the cross (marked by red rectangles) and the discontinuous areas. The ultimate unable switching ratio is about 31%, as calculated from the corresponding hysteresis loop, which is also displayed. The ultimate unable switching ratio includes the discontinuously (green rectangles) and continuously (red rectangles) scattered grey areas in Hall Bar. According to the previous works^[^ ^40^ ^]^ and stimulations in the Section S4 (Supporting Information), the observed domain wall motion and the presence of an unable switching area in the cross‐region can be explained in terms of a significant reduction of current density in the Hall cross‐region. Furthermore, the un‐driven scattering area can be understood in terms of the subtle AFM domains. The AFM domains do not have the same grain size, but rather there exists a distribution of their grain size. The large AFM domains are more stable than the small AFM domains.^[^ ^24^ ^]^ The stable domains cannot be switched by the SOT effect, and pin the adjacent Co atoms along the initial EB direction. An un‐switched discontinuous area is thus formed. On the other hand, the small domains may be metastable. They can be switched back and forth by the spin‐polarized current. These stable and metastable domains combined together are the cause of the asymmetric manipulation of the AFM interfacial states.

### 2.4 Dynamically Modulated Behaviors

**Figure 5:**
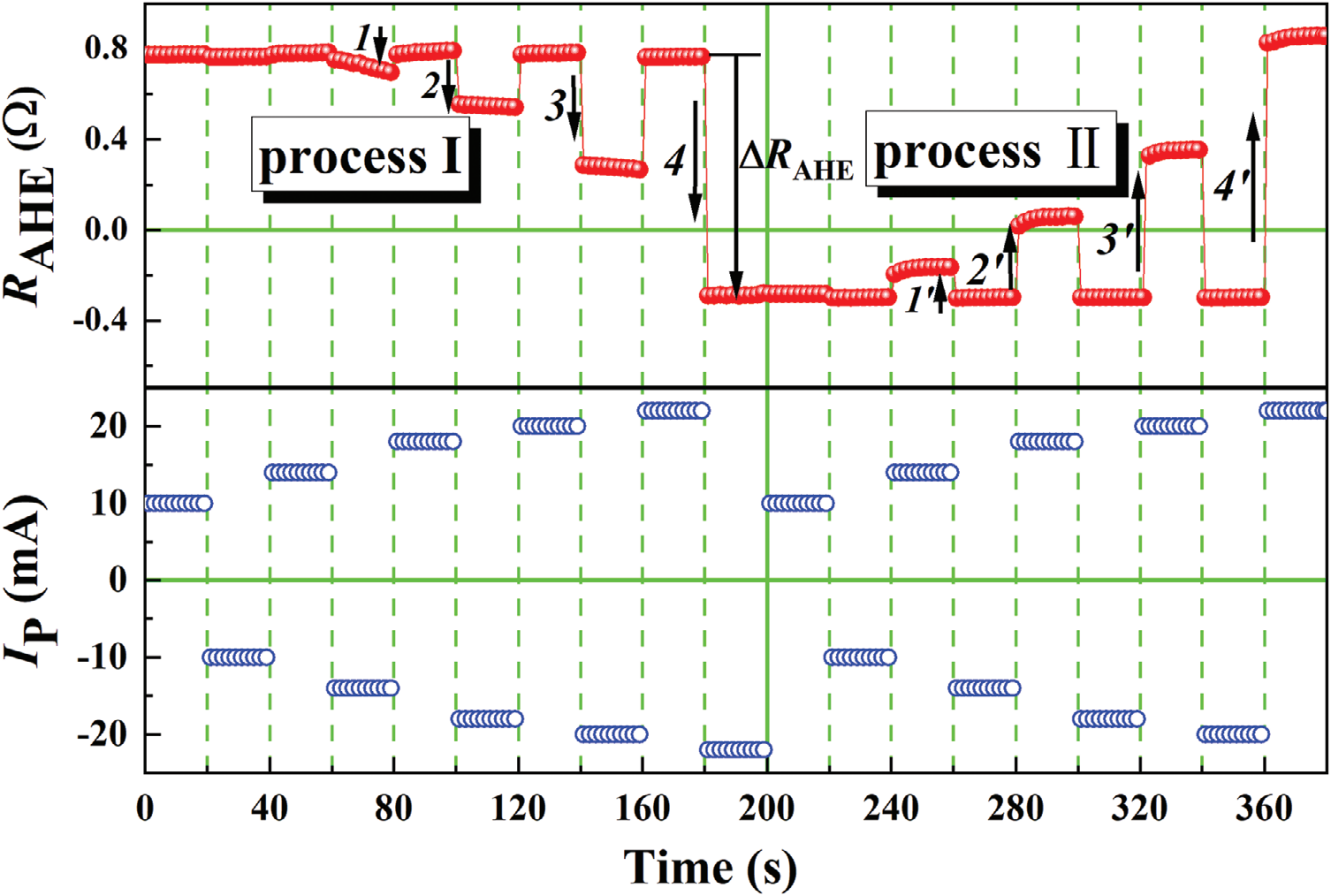
Dynamic modulating the magnetized switches by stepping increase negative/positive pulses from two different saturated initial states with IP field *H*_x_ fixed at 300 Oe.

In order to further check the stability of the SOT‐driven FM/AFM EB structure, dynamic and gradually increasing negative/positive pulses (∓10, ∓14, ∓18, ∓20, and ∓22 mA) are applied starting from the two sample states. **Figure** **5** shows the two processes of our experiments. In process **I**, the sample is modulated from the initial EB state; specifically, the EB field orients completely along the “up” direction. In process **II**, the sample is driven after the maximum negative pulse of −22 mA. According to Figure 3c, the initial state in process **II** is due to the presence of a small quantity of AFM domains pinned in the +*z*‐direction (the initial EB direction). It should be noted that except for the difference in the initial state, the sequences of applied pulses are the same for processes **I** and **II**. As shown in Figure 5, the *R*_AHE_ achieved with dynamically changing pulses is very stable. This provides strong evidence that SOT‐driven AFM materials can be well‐controlled and are suitable for applications. In addition, we also observed a subtle change of *ΔR*_AHE_ in steps 1 to 4 (in process **I**), which is lower than that in steps 1′ to 4′ (in process **II**). The reason is attributed to the part of AFM domains that are fixed and cannot be switched. As observed in the Kerr measurements, a fixed, un‐changeable EB field and magnetized regions in the +*z*‐direction exist throughout the sample. For process **II**, the SOT effect generated by a positive pulse switches the moments to the +z‐direction. The fixed unchanged EB field acts as an effective field along the +z‐direction to assist the reversal.^[^ ^41^ ^]^ Therefore, a current with a given amplitude can switch more magnetic moments in process **II**. This also demonstrates the possibility of asymmetrically manipulating the FM/AFM system and paves a new avenue for the realization of AFM‐spintronic devices. During the entire testing process, the total current repeated approximately 380 times without any significant instability or variation. This proves the stability of the asymmetric manipulation capabilities of our device under repeated testing.

### 2.5 Programmable Spin Logical Devices via the Asymmetric Manipulation of the AFM layer

Based on the above observations, the dependence of magnetization on the SOT‐driving EB exhibits a well‐defined asymmetric *R*_AHE_, which provides a significant opportunity to construct an AFM‐based spin logic device. AFM materials are immune to magnetic fields, and a large *H*_EB_ also makes the whole structure more stable against perturbations, which is good for application scenarios. Selecting FM/AFM heterostructure as the new spin logic cell could pave the way toward achieving high‐stability, anti‐interference, and logic‐in‐memory innovative spintronic architectures.

**Figure 6:**
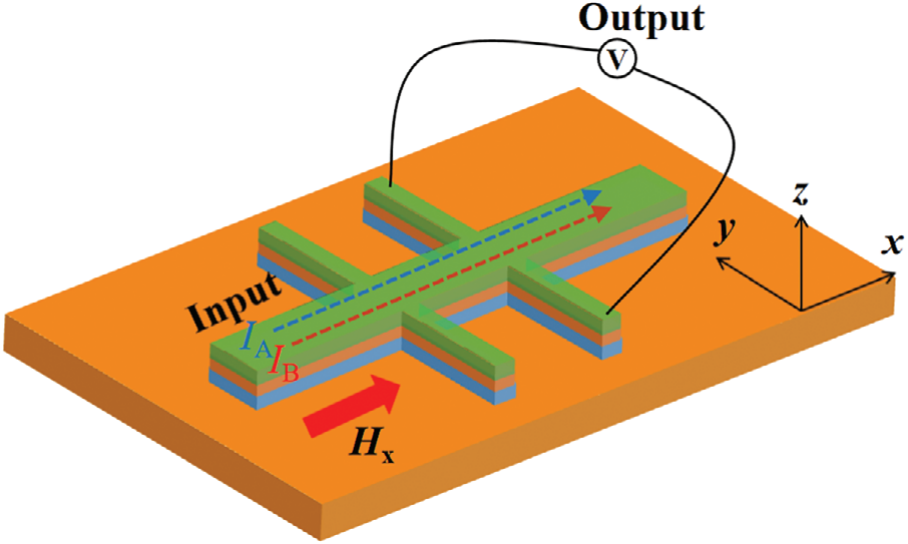
Spin logic cell based on Hall bar structure with test currents and picked‐up voltage.

A Hall bar structure was fabricated as the AFM‐based spin logic cell. Its schematic is shown in **Figure** **6**. To demonstrate its logic functionality, we first define *R*_AHE _= 0.8 Ω in the as‐deposited state as *R*_1_ and the *R*_AHE_ = −0.4 Ω of the state after application of *I*_p_ = −22 mA as *R*_2_. A determinant resistance *R*_th_ is defined as *R*_th_ = |*R*_2_‐*R*_1_|/2. *R*_th_ works as a threshold for identifying the logic output value. When the achieved *R*_AHE_ is larger than *R*_th_, the logic cell outputs “1”, while in the opposite case, it outputs “0”. The direction of the current pulse determines the constructed logic states, and the magnetic field is fixed at +300 Oe along the *x*‐axis. The final magnetization state that yields output is thus determined by the plus values of *I*_A_ and *I*_B_. Before performing each final Boolean logic operation, the prototype cell requires a processes of “**Reset**” or “**Set**” due to its non‐volatility. For “AND” and “OR” gates, the initial state should be set to *R*_2_ by a current pulse *I*_p_ of −22 mA. It works as a **Reset** process. For “NOT”, “NOR”, and “NAND” gates, a current pulse *I*_p_ of +22 mA is required to reach the state *R*_1_ state. It performs the **Set** process. **Figure** **7** demonstrates that five logic gates, namely “AND”, “OR”, “NOT”, “NOR”, and “NAND”, have been all realized in a single cell. Their truth tables are also listed to the side. We select an *I*_L_ of +11 or −11 mA for input “1”. An *I*_S_ of ±6 mA is applied in gates “AND” and “NAND” for input “0”, and an *I*_S_ of ±9 mA is applied for the other three gates for input “0”. In practice, as displayed by the green line in Figure 7, outputs “1” and “0” are identified by *R*_th_ ≥ 0.2 Ω and *R*_th_ < 0.2 Ω, respectively.

Figure 7Logic process of the spin logic device. Input current pulse *I*_L_, *I*_S_ under *H*_x _= 300 Oe and output *R*_AHE_ in a) “AND,” b) “OR,” c) “NAND,” d) “NOR,” and e) “NOT” gates, respectively, as well as their corresponding truth tables.

**Figure:**
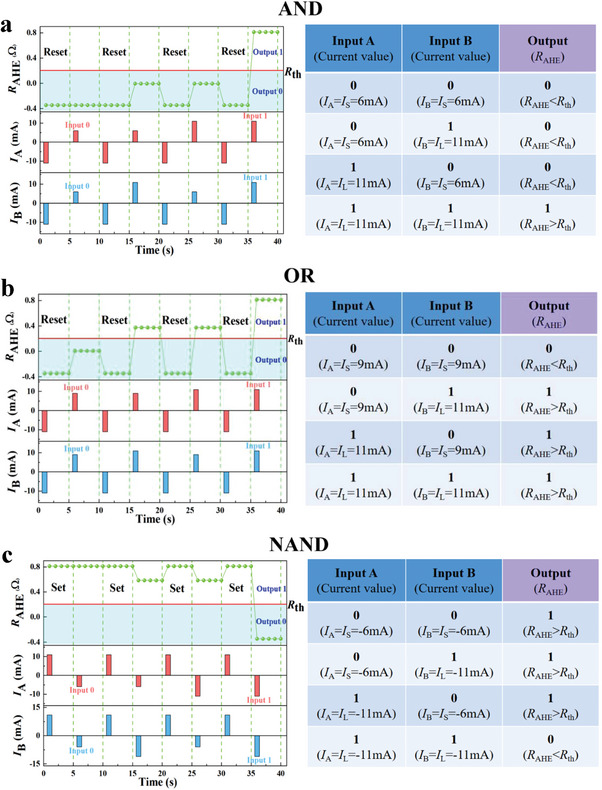

**Figure:**
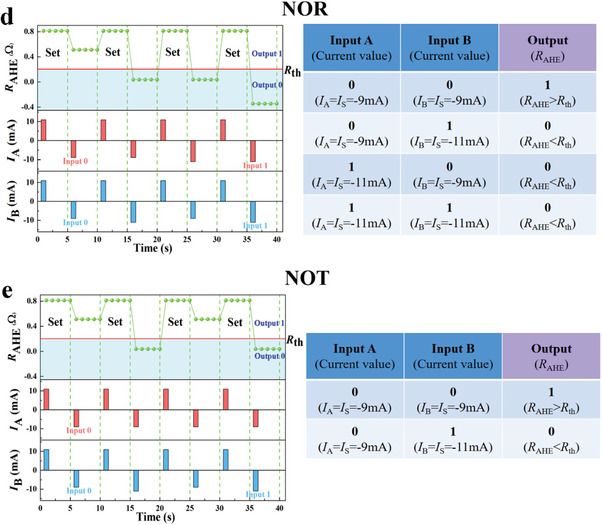

For the “AND” gate shown in Figure 7a, the **Reset** process is first applied to switch the EB field and the magnetization of the Co layer to the ‐*z*‐axis. In the logic operation, an *I*_L_ of 11 mA and an *I*_S_ of 6 mA are adopted for inputs “1” and “0”. Only if A = B = 1 or *I*_A_ = *I*_B_ = 11 mA, the EB field and the magnetic moments of the Co layer can be switched to the “up” state, and the cell outputs logic “1”. If either input A or B is chosen as “0”, for example, *I*_A_ = 11 mA and *I*_B_ = 6 mA, an *I*_A_+*I*_B_ of 17 mA is too low to achieve an *R*_AHE_ larger than *R*_th_, and then the cell outputs logic “0”. In this case, the device functions as an “AND” gate.

For an “OR” gate, as shown in Figure 7b, a prior **Reset** process is also required to reach the Boolean logic state. *I*_L_ and *I*_S_ are 11 and 9 mA, respectively. Except for A = B = 0 or *I*_A_ = *I*_B _= 9 mA, other combinations can all result in *I*_A_+*I*_B_ ≥ 20 mA and thus switch the magnetic moments to the “up” state, which yields *R*_AHE_ > *R*_th_ and the device functions as an “OR” gate.

For a “NAND” gate as shown in Figure 7c, a **Set** process is adopted to initialize the cell. In the logic operation, an *I*_L_ of −11 mA and an *I*_S_ of −6 mA are adopted for inputs “1” and “0”. In this case, only if A = B = 1 or *I*_A_ = *I*_B_ = −11 mA, an *I*_A_+*I*_B_ of −22 mA can switch the device to “0”. The logic output remains “1” for other inputs.

For a “NOR” gate, as shown in Figure 7d, we set *I*_L_ = −11 mA and *I*_S_ = −9 mA, initializing the device by the **Set** process to *R*_1_. When A and B input “0”, according to Figure 3a, a total pulse of −18 mA can only switch a small proportion of the moments. Then, the device also displays a resistance larger than *R*_th_. It outputs “1”. Otherwise, the device outputs “0”.

For a “NOT” gate shown in Figure 7e, we set *I*_L_ = −11 mA and *I*_S_ = −9 mA, initializing the device by the **Set** process. The input A is set to be “0”. After the initialization, the magnetic moments combined with the EB field are oriented in the “up” state. When the B inputs “0”, the total driving current is only −18 mA. According to Figure 3b, this current amplitude can only reverse a few parts of moments to the “down” state, and the vast majority of the moments are still oriented in the “up” state, which results in a slight decrease in *R*_AHE_ compared with the *R*_1_ state. Therefore, the device in this case outputs “1”, and vice versa, it outputs “0”.

Notably, compared with HM/FM/oxide structures used for realizing spin logic functions,^[^ ^42^, ^43^, ^44^ ^]^ exploiting the asymmetric manipulation of the Pt/Co/IrMn structure does not require tuning the current pulse and the lateral magnetic field simultaneously, and five logic gates can be realized in a single cell. Furthermore, the logic gate can also function through the manipulation of the lateral magnetic field *H*_x_ rather than the current pulse *I*_p_. By adopting free‐field control methods, such as wedge structures^[^ ^45^ ^]^ and interlayer exchange coupling,^[^ ^46^ ^]^ the asymmetric manipulation of AFM interfacial moments to control the EB field and generate different remanence states at zero field will pave the way for the construction of novel spin logic devices.

## 3 Conclusion

In summary, our study presents a deterministic magnetization reversal process achieved via SOT based on a Pt/Co/IrMn perpendicular EB structure. The ability to manipulate the magnetization and EB field via SOT is demonstrated by changing the current direction and amplitude of the current pulse *I*_p_ with a fixed IP magnetic field *H*_x_. We show that current pulses with the same amplitude but opposite directions have asymmetric manipulation capabilities. This is attributed to the un‐switchable AFM domains and the current distribution influenced by the Hall bar structure. In addition, five spin logic gates are realized in a single cell controlled by two different input currents and an IP magnetic field. The control of the magnetic moments in the HM/FM/AFM system provides the possibility to use AFM materials in logical operations and neuromorphic computing.

## 4 Experimental Section

### Sample Growth

Thin stacked structures consisting of Ta (1 nm)/Pt (3 nm)/Co (*t*_Co_)/Ir_20_Mn_80_ (5 nm)/Ta (1.5 nm) with *t*_Co_ ranging from 0.6 to 1.7 nm were deposited on thermally oxidized Si substrates using magnetron sputtering. The thin bottom and top Ta layers served as adhesion and capping layers, respectively. The multiplayer films were deposited at room temperature. Throughout the deposition, the base pressure was 2 × 10^−8^ Torr, and the sputtering power for the whole structures was 30 W, with an Ar pressure of 3 mTorr. To electrically switch the moments at the FM/AFM interface and detect such variation, the as‐deposited films were patterned into 10 µm‐wide and 200 µm‐length Hall bars using standard photolithography and Ar‐ion etching.

### Current Pulses Application

A SOT‐driven pulse combined with a small sampling current to pick up the Hall voltage is applied to probe the stable nonvolatile magnetization state. The SOT‐driven pulse is first applied and released. Then, after an interval time of 100 ms, a subsequent pulse with an amplitude of 5 µA and a duration of 5 ms is adopted to pick up the Hall voltage.

## Conflict of Interest

The authors declare no conflict of interest.

## Supporting information

Supporting Information

## Data Availability

The data that support the findings of this study are available from the corresponding author upon reasonable request.
