# Supplementary material for: Asymmetric Manipulation of Perpendicular Exchange Bias and Programmable Spin Logical Cells by Spin–Orbit Torque in a Ferromagnet/Antiferromagnet System
Source: Adv Sci (Weinh). 2024 Jul 10;11(34):2403648. doi: 10.1002/advs.202403648 (PMC11425839; doi:10.1002/advs.202403648)
Supplement: Supplementary file 1 — Supporting Information [file ADVS-11-2403648-s001.pdf]

ADVANCED SCIENCE
Open Access

# Supporting Information

for *Adv. Sci.*, DOI 10.1002/advs.202403648

Asymmetric Manipulation of Perpendicular Exchange Bias and Programmable Spin Logical Cells by Spin–Orbit Torque in a Ferromagnet/Antiferromagnet System

*Lei Guo, Guopeng Shi, Guocai Wang, Hua Su, Huaiwu Zhang and Xiaoli Tang**

Supplementary Materials for

# Asymmetric manipulation of perpendicular exchange bias and programmable spin logical cells by Spin–Orbit Torque in an ferromagnet/antiferromagnet system

Lei Guo[1], Guopeng Shi[1], Guocai Wang[1], Hua Su[1], Huaiwu Zhang[1], and Xiaoli Tang[1]*

[1]*State Key Laboratory of Electronic Thin Films and Integrated Devices, School of Electronic Science and Engineering, University of Electronic Science and Technology of China, Chengdu 611731, China*

Corresponding authors.Email: tangtang1227@uestc.edu.cn

## 1. The influence of IP magnetic field $H_x$ to $R_{AHE}$

The magnetization switching direction controlled by SOT depends on $H\times\sigma$, which $\sigma$ and $H$ refer to spin polarization and effective in-plane field including $H_x$, respectively. [1,2] Figure S1 shows the influence of reversing IP magnetic field $H_x$ to $R_{AHE}$. Figure S1a illustrates that the dependence of $R_{AHE}$ on applied current $I_p$ with $H_x$=-300 Oe is similar to that with $H_x$=+300 Oe. $R_{AHE}$ with the same $I_p$ and opposite $H_x$ has opposite switching direction only, as shown in Figure S1b. These results are consistent with the SOT-driven the theory.

What's more, Figure S1c shows the $R_{AHE}$ loops versus $H_z$ in different positive IP magnetic fields. When the external magnetic field reaches or exceeds 300 Oe, the magnetic moment switching of the Co layer no longer increases in either positive or negative directions. So, we have chosen 300 Oe as the magnitude of the assistant field.

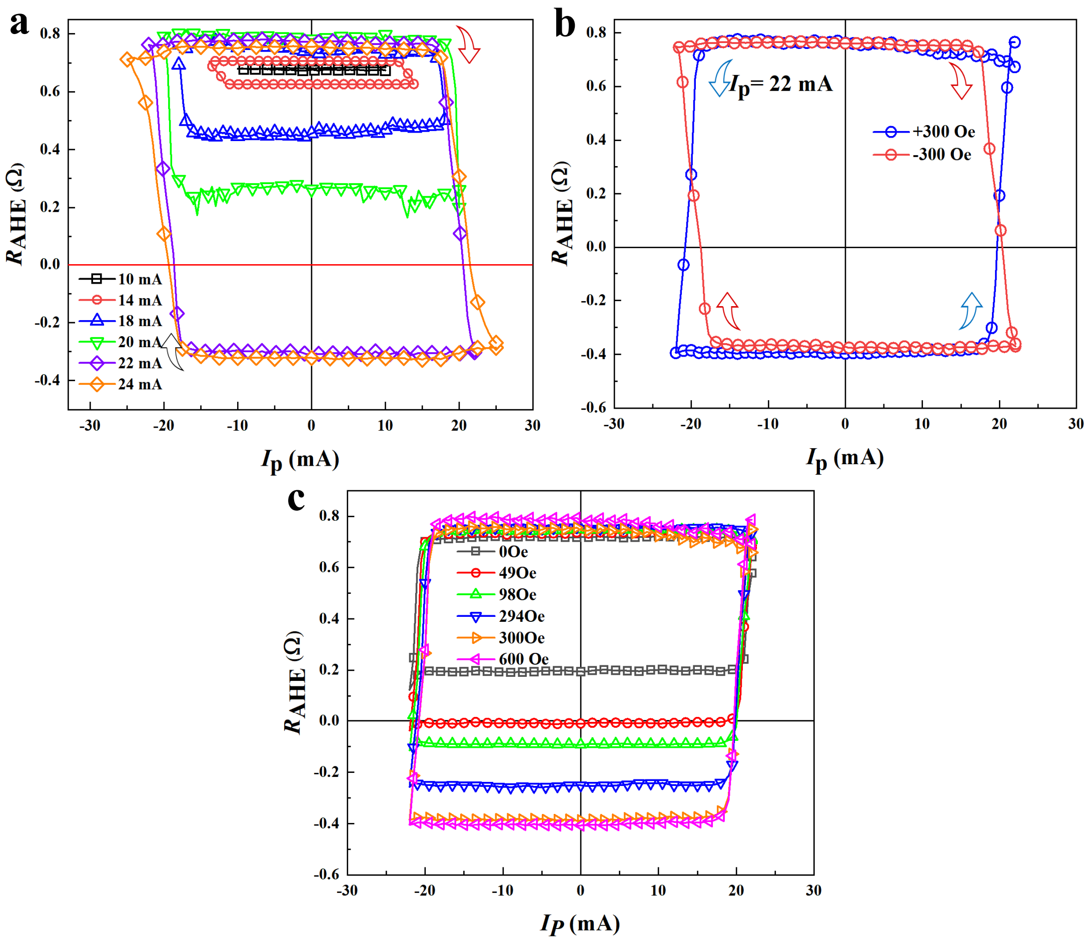


**Figure S1.** The influence of IP magnetic field $H_x$ to $R_{AHE}$. a) $R_{AHE}$ versus $I_p$ curves with varying $I_p$ and $H_x$=-300 Oe. b) $R_{AHE}$ versus $I_p$ curves with positive and negative $H_x$. c) $R_{AHE}$ versus $I_p$ curves with different positive $H_x$.

## 2. The $R_{AHE}$ loops versus $H_z$ of Ta/Pt/Co/Ta without AFM layer in positive and negative IP magnetic fields

Exchange bias roots in the exchange coupling effect generated by the unmatched magnetic moments between the Co/IrMn interface, leading to the deflection in the hysteresis loop along the magnetic field axis. [3] If there is no AFM layer in the HM/FM system, there is no EB field existing in the system. The $R_{AHE}$ loops versus $I_P$ of Ta (1nm)/Pt (3 nm) /Co (1.4 nm)/Ta (1.5 nm) with current pulse in positive and negative IP magnetic fields, as shown in Figure S2, are almost symmetric about the y-axis with no EB field. It is completely different with the HM/FM/AFM structure.

While the directions of the IP magnetic field do not influence the switching degree of the magnetization, only inversing the switching direction, which is similar to the performance of Pt/Co/IrMn structure.

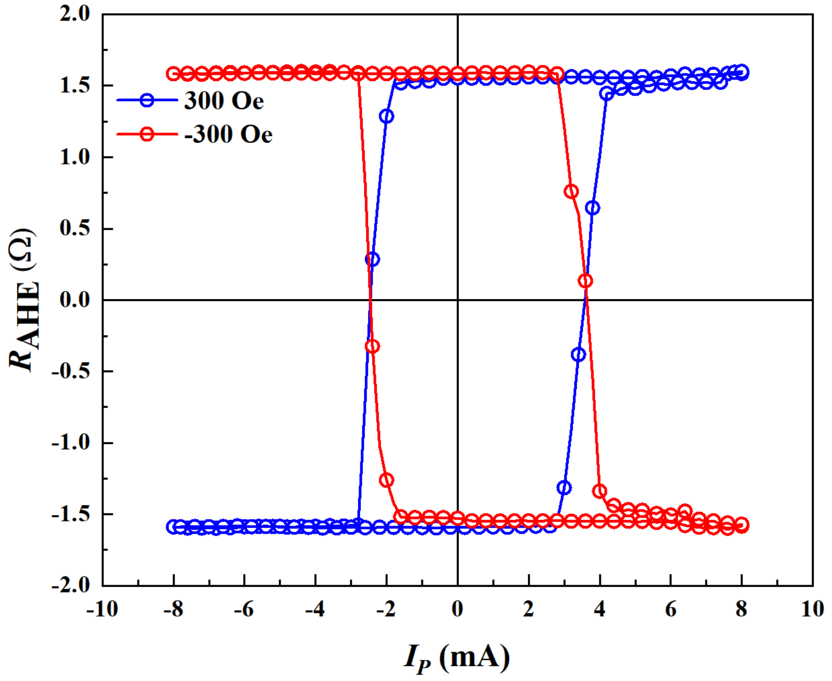


**Figure S2.** $R_{AHE}$ versus $I_P$ curves of Ta (1 nm)/Pt (3 nm)/Co (1.4 nm)/Ta (1.5 nm) with ±300 Oe magnetic field .

### 3. The effect of Joule heating on the switching of the IrMn interfacial spin

To verify whether the switching at the IrMn interface is caused by the Joule heating induced by the applied current, which raises the temperature of IrMn beyond its blocking temperature ($T_B$), we have estimated the temperature rise of IrMn after applying current pulses based on the previous researches. [4,5] As shown in Figure S3a, we have measured the change of the longitudinal resistance ($\Delta R_{XX}$) of the sample from 300K to 450K. Then we measured the $\Delta R_{XX}$ of the sample under current pulses ranging from 0 to 22 mA in Figure S3b. Compared with the correlation between $\Delta R_{XX}$ and temperature in Figure S3a, it can be concluded that the maximum temperature rise

of the sample under a 22mA current pulse is approximately 40K.

To measure the $T_B$ of the IrMn layer, the sample was heated on a heating plate, and the $H_{EB}$ at different temperatures were achieved. As shown in Figure S3c, the $H_{EB}$ gradually decreases with increasing temperature and drops to 0 around 440K. This indicates that the $T_B$ of IrMn is approximately 440K. Therefore, the $T_B$ of the IrMn layer is significantly higher than the temperature rise induced by the current pulse. Thus, the effect of Joule heating on the spin switching at the IrMn interface can be excluded.

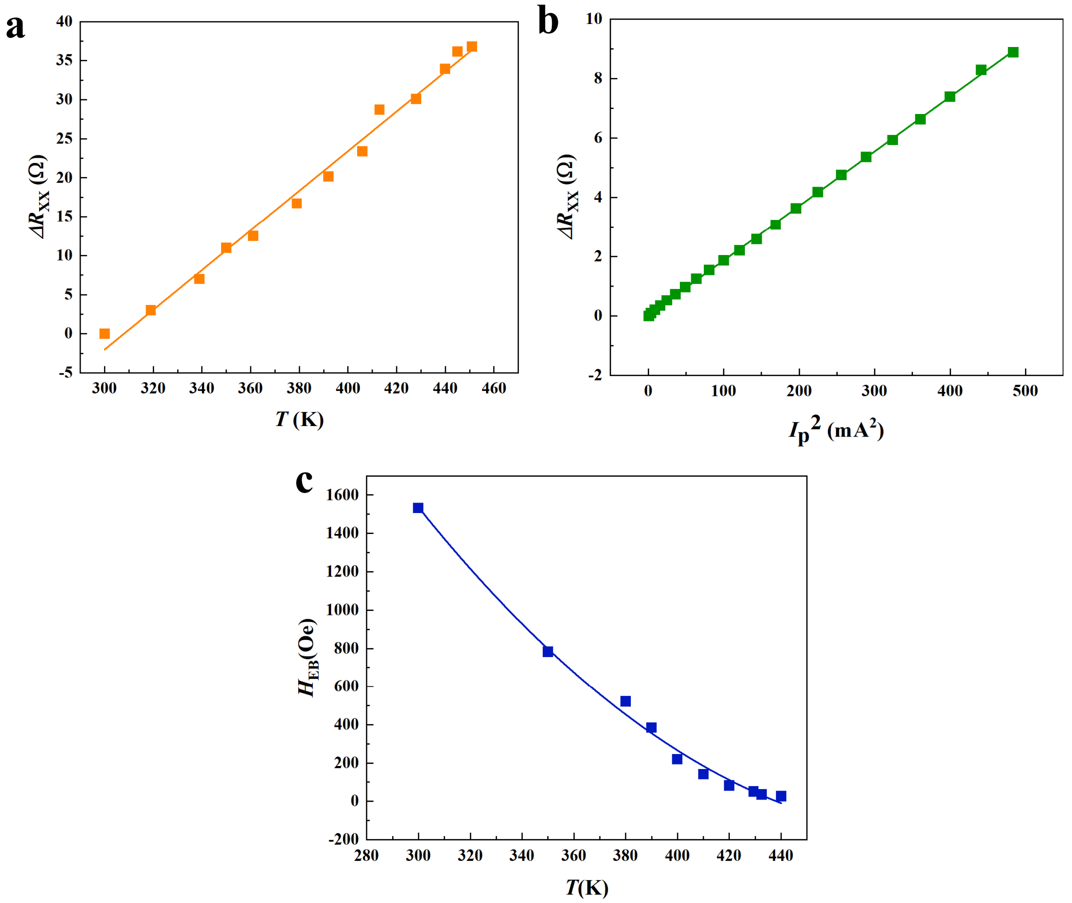


**Figure S3.** Testing of the temperature increase due to Joule heating during the current pulse and the blocking temperature of the IrMn layer. a) The change of the longitudinal resistance ($\Delta R_{XX}$) as a function of temperature. b) $\Delta R_{XX}$ measured during the current pulse. c) $H_{EB}$ varies with temper-

ature. The $T_B$ of IrMn in the manuscript is approximately 440K.

## 4. The current density distribution in the Pt layer in the Hall Bar using COMSOL simulations

During the process of SOT switching, the magnetization reversal of Hall cross is uneven. We examined the current density distribution in the Pt layer in the Hall Bar using COMSOL simulations. When the current pulse is equal to the critical current 18 mA, the current density in Pt layer is about $J_0$ = 1.59×10$^7$ A/cm$^2$. Figure S4 shows the current density distribution with different current pulse. Different colors respond to different current densities while the chromatic aberration reflects the difference of current densities.

For $I_p$ =-18 mA (Figure S4b), there large areas with current density lower than $J_0$, indicating the un-switchable magnetization. Nevertheless, the areas with low current density reduce following the increscent $I_p$ with current density higher than $J_0$ covering more and more areas, which means the switched areas expand (Figure S4c and Figure S4d). While for $I_p$ =-14 mA (Figure 4a), most areas of the Hall cross region have a current density lower than $J_0$. Therefore, less areas can be switched. The variation of these areas generally agrees with the change of Hall cross in Figure 4. Therefore, the uneven current density distribution can affect the magnetization reversal to some extent.

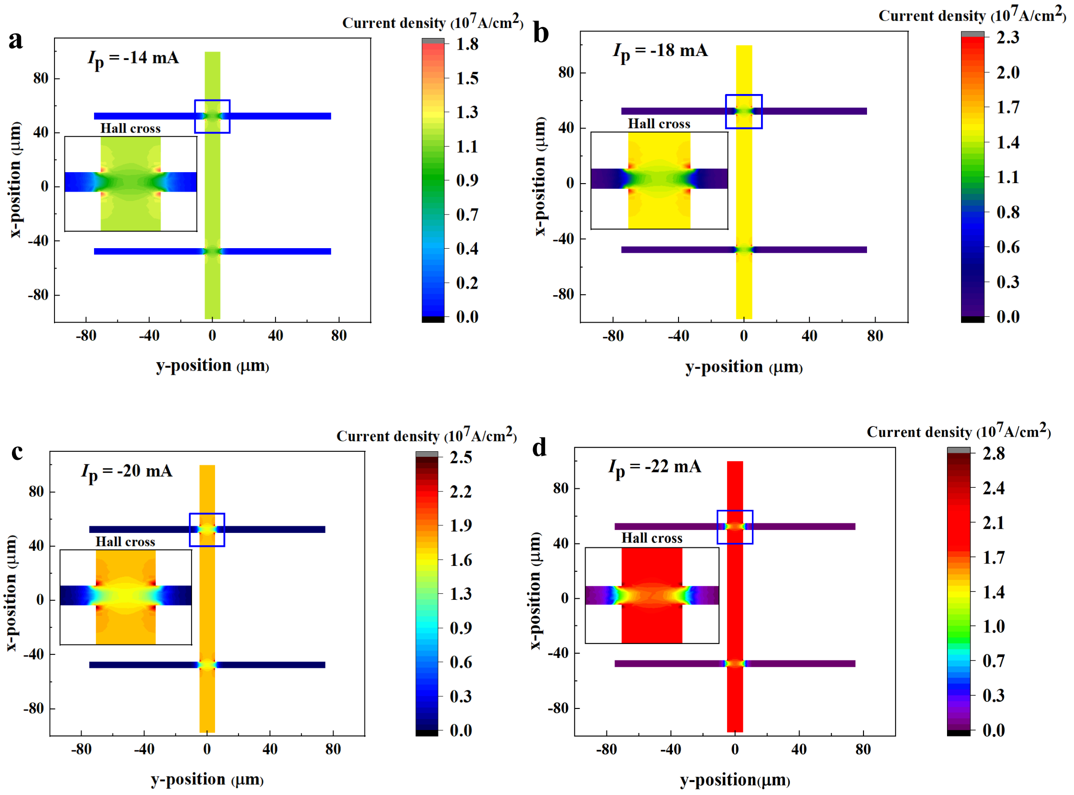


**Figure S4.** Current density distribution in the Pt layer in the Hall Bar. a) -14 mA. b) -18 mA. c) -20 mA. d) -22 mA.


[1] H. Bai, Y. C. Zhang, L. Han, Y. J. Zhou, F. Pan, C. Song, *Appl. Phys. Rev.* **2022**, *9*, 041316.

[2] Y. C. Lau, D. Betto, K. Rode, J. M. D. Coey, P. Stamenov, *Nat. Nano.* **2016**, *11*, 758.

[3] J. Nogues, I. K. Schuller, *J. Magn. Magn. Mater.* **1999**, *192*, 203.

[4] E. Z. Zhang, Y. C. Deng, X. H. Liu, X. Z. Zhan, T. Zhu, K. Y. Wang, *Phys. Rev. B* **2021**, *104*, 134408.

[5] P. H. Lin, B. Y. Yang, M. H. Tsai, P. C. Chen, K. F. Huang, H. H. Lin, C. H. Lai. *Nat. Mater.* **2019**, *18*, 335.
